# Superior visual rhythm discrimination in expert musicians is most likely not related to cross-modal recruitment of the auditory cortex

**DOI:** 10.3389/fpsyg.2022.1036669

**Published:** 2022-10-20

**Authors:** Maksymilian Korczyk, Maria Zimmermann, Łukasz Bola, Marcin Szwed

**Affiliations:** ^1^Intitute of Psychology, Jagiellonian University, Kraków, Poland; ^2^Institute of Psychology, Polish Academy of Sciences, Warszawa, Poland

**Keywords:** neuroplasticity, attention, inferior parietal lobe, musicians, fMRI

## Abstract

Training can influence behavioral performance and lead to brain reorganization. In particular, training in one modality, for example, auditory, can improve performance in another modality, for example, visual. Previous research suggests that one of the mechanisms behind this phenomenon could be the cross-modal recruitment of the sensory areas, for example, the auditory cortex. Studying expert musicians offers a chance to explore this process. Rhythm is an aspect of music that can be presented in various modalities. We designed an fMRI experiment in which professional pianists and non-musicians discriminated between two sequences of rhythms presented auditorily (series of sounds) or visually (series of flashes). Behavioral results showed that musicians performed in both visual and auditory rhythmic tasks better than non-musicians. We found no significant between-group differences in fMRI activations within the auditory cortex. However, we observed that musicians had increased activation in the right Inferior Parietal Lobe when compared to non-musicians. We conclude that the musicians’ superior visual rhythm discrimination is not related to cross-modal recruitment of the auditory cortex; instead, it could be related to activation in higher-level, multimodal areas in the cortex.

## Introduction

It is well established that training for a highly demanding skill can increase several cognitive abilities (i.e., Schellenberg and Weiss, 2013) and reorganize the structure of the brain (i.e., Sampaio-Baptista and Johansen-Berg, 2017). While most studies concentrate on cognitive enhancement within the trained sensory modality, several notable studies have also explored how expertise in one modality influences sensory functions in another modality (Huang et al., 2010; Bouhali et al., 2020; Heimler and Amedi, 2020). Professional musicians are a very valuable subject group for studying plasticity at the behavioral and neuronal levels (Herholz and Zatorre, 2012; Olszewska et al., 2021). Such studies on musical expertise can provide insight into the inter-sensory transfer of highly trained cognitive abilities. Some aspects of music, such as pitch and timbre, are not easily transferable to other senses. However, temporal features of music, such as duration and rhythm, can be presented not only in auditory, but also in visual or haptic modalities (Kosonen and Raisamo, 2006; Grahn, 2012). Studying rhythm perception provides an opportunity to explore the cross-modal aspects of behavioral enhancement.

Despite its amodal nature, rhythm is an aspect of music primarily associated with auditory experience. This observation is reflected in the advantage of the auditory system over other sensory systems in processing temporal information (e.g., Fendrich and Corballis, 2001; Repp and Penel, 2002). Many studies report a higher average accuracy level across auditory timing tasks compared to visual timing tasks (i.e., Grahn et al., 2011; Grahn, 2012). However, training in auditory processing of rhythm can lead to improved performance in other modalities. Thus, Barakat et al. (2015) showed that the ability to distinguish two series of rhythms presented in the visual modality can significantly improve after short-term auditory or audio-visual training. Professional musicians are known to exhibit higher performance in visual rhythm processing (Rammsayer and Altenmüller, 2006; Rammsayer et al., 2012).

The neural mechanisms by which auditory training may lead to enhanced visual processing remain unclear. Here, we hypothesize that the behavioral improvement in the visual domain arises from cross-modal recruitment of the auditory cortex. This would suggest that the auditory cortex in highly trained musicians is recruited for auditory and visual rhythm-related tasks.

This hypothesis is based on the concept of task-specific sensory independent organization of the cortex (i.e., Amedi et al., 2017; Heimler and Amedi, 2020), and proposes that during reorganization the cerebral cortex preserves its functions (e.g., reading or object recognition), regardless of the modality in which these tasks are performed (i.e., Heimler et al., 2014). For example, tactile reading induces activation in the visual cortex in the blind (Reich et al., 2011), and visually presented rhythm recruits the auditory cortex in the deaf (Bola et al., 2017). Cross-modal reorganization is possible outside sensory-deprived populations after intensive training. Siuda-Krzywicka et al. (2016) showed that sighted Braille readers recruit their ventral visual cortex for tactile Braille reading after 9 months of training. Moreover, in sighted subjects the lateral occipital complex is engaged in shape processing presented in a different modality (Amedi et al., 2001; Kim and Zatorre, 2011), and the occipito-temporal region hMT/V5 is activated during auditory and visual motion-direction tasks (Rezk et al., 2020). Cross-modal activation has been found in the visual cortex of sighted users of sensory substitution devices (Amedi et al., 2007), and in expert Mah-Jong players (Saito et al., 2006).

In parallel, massive reorganization in the auditory cortex of musicians has been observed in several reports (Schneider et al., 2002; Aydin et al., 2005; Pantev and Herholz, 2011). de Manzano and Ullén (2018), for example, it was found that monozygotic twins who performed music had greater cortical thickness in the auditory and motor regions than those who did not practice music. Multisensory tasks where visual, tactile and somatosensory stimuli are used are known to induce activation in the higher auditory cortex in professional musicians (Pantev et al., 2015). Hoenig et al. (2011) reported activation in the auditory association cortex induced by musical instruments presented visually only to expert musicians. Finally, Haslinger et al. (2005) observed activation in the auditory areas of professional pianists who watched silent video clips of piano playing. In general, these studies suggest that musical training can increase the responsiveness of the auditory cortex to music-related information, even when this information is presented in the visual or tactile modality.

With these studies in mind, we set out to investigate whether extensive musical training in the auditory modality can lead to similar cross-modal and task-specific recruitment of the auditory cortex for rhythm perception. If confirmed, this would further support the view that cross-modal task-specific plasticity of the brain can be conceived as a general principle that applies to brain reorganization in multiple contexts.

## Materials and methods

### Participants

Thirty-eight participants (18 professional musicians; 20 non-musicians) enrolled in the experiment. Musicians (the experimental group; all pianists; 13 women; mean age 22.8, SD = 3.7; the average length of education 14.9, SD = 2.5) had more than 10 years (*M* = 13.8 years, SD = 2.5) of formal training on a musical instrument. All started playing the piano between the ages of 3 and 8. Non-musicians (the control group; 14 women; mean age 23.5, SD = 2.7; the average length of education 14.9, SD = 2.0) had no musical training and did not play a musical instrument. Musicians and non-musicians were matched for sex, age, and years of education (all *p* > 0.49). All participants were right-handed; they had normal or corrected-to-normal vision and no neurological deficits. The Committee for Research Ethics of the Institute of Psychology of Jagiellonian University approved the research described in this article. Written informed consent was obtained from all participants.

### Outline of the experiment

**Figure 1:**
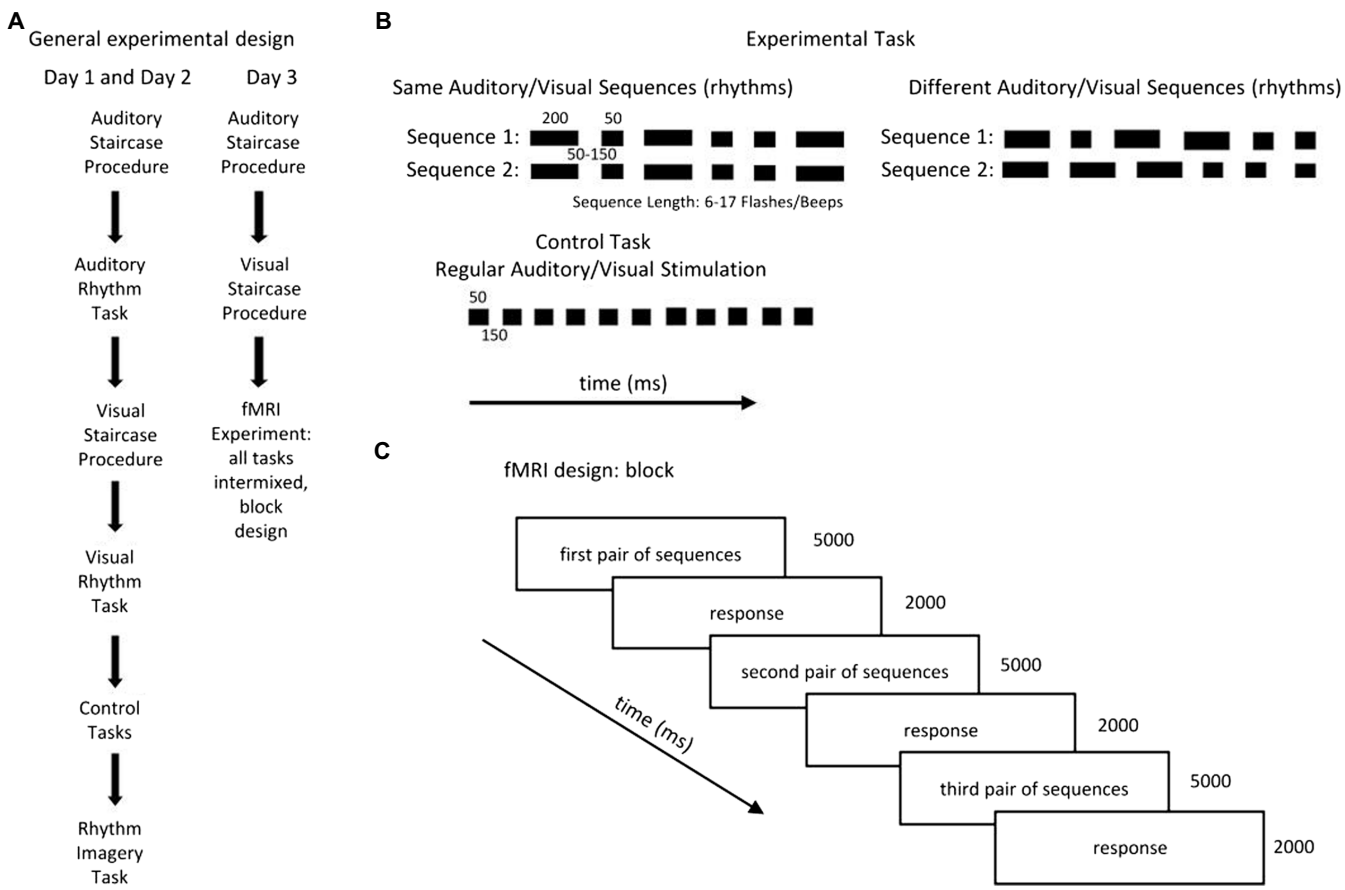
**(A)** The subjects performed three types of tasks: a rhythm discrimination task (the experimental tasks: Auditory Rhythm and Visual Rhythm), which was adapted from Bola et al. (2017); a control task (Auditory Control and Visual Control); and a task in which the participants were asked to imagine rhythmical patterns (Rhythm Imagery Task). The experiment took place over three consecutive days. On the first and second day, the subjects performed all tasks separately, always in the same order. **(B)** The experimental tasks consisted of two sequences with the same number of beeps/flashes of short (50-ms) and long (200-ms) duration, separated by SO to 150-ms blank intervals. Participants were asked to determine whether the pairs of sequences were the *same* or different. In the control’ conditions, subjects listened to/watched passive sequences of beeps/flashes presented at a constant pace [stimuli (50 ms) separated by blank intervals (150 ms)]. In the Rhythm Imagery task, subjects had to imagine rhythmical sequences. **(C)** The fMRI experiment consisted of three runs in which all tasks (Auditory Rhythm, Visual Rhythm, Auditory Control, Visual Control, and Rhythm Imagery) were intermixed. In all runs, each task was repeated 15 times and was presented in a block of three pairs of sequences. Total duration of the blocks was 21 s; the rest period between blocks was 8, 10, or 12 s.

The subjects performed three types of tasks: a rhythm discrimination task: 1. the experimental tasks: Auditory Rhythm and Visual Rhythm, which was adapted from Bola et al. (2017), 2. a control task (Auditory Control and Visual Control), and 3. a control task in which the participants were asked to imagine rhythmical patterns (Rhythm Imagery; Figures 1A,B).

The experiment took place over three consecutive days (Figure 1A). Every day, subjects performed adaptive staircase procedures in the auditory and visual domains to determine their performance levels. On the first and second days, the subjects performed the Auditory Staircase Procedure, the Auditory Rhythm task, the Visual Staircase Procedure, the Visual Rhythm task, the Auditory Control task, the Visual Control task, and the Rhythm Imagery task (Figure 1A) outside of the scanner, always in the same order. On the third day, the subjects performed the Auditory and Visual Adaptive Staircase procedures and the fMRI experiment (Figure 1A).

The Adaptive Staircase Procedure was used to control the level of participants’ ability to perform the experimental tasks in both modalities. It was identical to the procedure used by Bola et al.’ (2017). The length of the sequences was adjusted to the individual subject’s performance. The participants began the Staircase Procedure with sequences of six beeps/flashes. The sequence length increased by one item after a correct answer (upper limit of the sequence length = 17 beeps/flashes). If subjects made a mistake, the number of beeps/flashes decreased by two items (lower limit of the sequence length = 6 beeps/flashes). The presentation pace was manipulated accordingly by changing the durations of the blank intervals between visual and auditory stimuli (6–8 flashes/beeps in the sequence: 150 ms; 9–11 flashes/beeps in the sequence: 100 ms; 12 flashes/beeps in the sequence: 80 ms; 13–17 flashes/beeps in the sequence: 50 ms). The procedure ended after six incorrect answers. The procedure was identical in Auditory and Visual experimental tasks.

The number of items obtained in the visual staircase was identical for all participants, in both groups (*n* = 6, the lower limit). The average number of items obtained in the auditory staircase was 13 items (SD = 1.32) for musicians and 12 items (SD = 2.50) for controls and did not significantly differ across groups [*t*(36) = 1.45, *p* = 0.16].

On the first 2 days (Figure 1A), following the Auditory Adaptive Staircase Procedure, subjects completed 60 trials of the Auditory Rhythm Task. Then, following the Visual Adaptive Staircase Procedure, they completed 60 trials of the Visual Rhythm Task. After that, they performed six trials of the Auditory Control Task and Visual Control Task. Finally, they performed 12 trials of the Rhythm Imagery Task.

On the third day (Figure 1C), after the Auditory and Visual Adaptive Staircase Procedures, participants performed the fMRI experiment. The fMRI experiment consisted of three runs in which all tasks (Visual Rhythm, Auditory Rhythm, Visual Control, Auditory Control, and Rhythm Imagery) were intermixed in a pseudorandom order. Each task was presented in a block of three pairs of sequences. Each block type was repeated five times per run. In summary, each task throughout the fMRI experiment was repeated 45 times. The total duration of the blocks was 21 s and the rest period between the blocks was variable and lasted 8, 10, or 12 s. In all tasks, participants had their eyes open and focused on a small circle in the center of the screen.

### Stimuli

In the Auditory Rhythm Task and the Auditory Control Task, the subjects heard tonal beeps (360 Hz, ~60 dB) binaurally on headphones; they had to look at a small, bright, circle on a dark-gray background (diameter: 0.1°; luminance: 68 cd/m^2^). The Visual Rhythm Task and the Visual Control Task consisted of small bright flashing circles on a dark-gray background (diameter: 3°; mean display luminance: 68 cd/m^2^). During behavioral testing, flashes were shown on a laptop screen, during fMRI, on the scanner display *via* a mirror (32-inch HD LCD monitor; 60-Hz refresh rate).

Auditory and Visual Rhythm tasks (Figure 1B) were composed of two sequences with the same number of flashes/beeps of a short (50-ms) and long (200-ms) duration. Blank intervals were presented between individual flashes/beeps, and the duration of each blank interval was randomly chosen from three possible values (50 ms, 100 and 150 ms). The sequence pairs presented to the subjects were identical (e.g., long-short-short-long-short-long vs. long-short-short-long-short-long) or different (e.g., long-short-short-long-short-long vs. long-long-short-short-short-long). In the different condition only the first and last flashes/beeps were the same in both sequences. The interval between the sequences lasted 2 s. In Auditory and Visual Rhythm tasks as, well as in the staircase procedure, participants were asked to judge whether the two sequences were the same or different and after the second sequence, they had to press the corresponding button within 2 s. In the control tasks (Figure 1B) in both modalities, participants were asked to watch/listen to the same flashes/beeps presented at a constant pace (50 ms separated by 150-ms blank intervals). After that, they had to press any response button when the question mark appeared.

In the Rhythm Imagery Task, after seeing a white cloud (presented for 2 s), the participants had to imagine a visually-presented rhythm, similar to the ones presented in the Visual Rhythm task, for 7 s while an empty dark gray background was being presented. The rhythm imagery condition was meant as a control condition in case we obtained a main positive result. It was meant to exclude the possibility that putative activations in the auditory cortex induced by the visual rhythm discrimination task could be driven exclusively by imagery. Since we found no activations in the auditory cortex for the visual rhythm condition in the first place, we decided not to dwell on the imagery condition and not to report the results obtained in this condition in this paper.

All tasks were presented using Presentation (Neurobehavioral Systems).^1^ Sequences of visual and auditory rhythms were generated randomly in the staircase procedures, the behavioral training procedures, and the fMRI experiment. Since it is unlikely that the same visual/auditory rhythm sequence was presented more than once to the same subject, any behavioral effects reported are unlikely to be driven by subjects remembering and recalling specific sequences.

### fMRI data acquisition

All fMRI data were acquired at Małopolskie Centrum Biotechnologii in Kraków. Functional MR scans were collected using an EPI sequence on a 3 T Siemens Skyra scanner. A 64-channel head coil was used (flip angle = 90°; TR = 2,000 ms; TE = 26 ms; FOV = 192 mm; 64 × 64 matrix). 37 contiguous axial slices (thickness 3.0 mm; in-plane resolution = 3.0 × 3.0 mm^2^) were collected. For anatomical reference and spatial normalization, T1-weighted images were acquired using an MPRAGE sequence (176 slices; FOV = 256 mm; TR = 2,300 ms, TE = 2.98 ms, voxel size = 1 × 1 × 1 mm).

### Behavioral data analysis

Behavioral data was analyzed using SPSS 22 (SPSS Inc.).^2^ A three-way repeated-measures ANOVA (group × modality × day) was used to compare the level of accuracy on each experimental day to check the two effects: the effect of training and the between-group effect. Bonferroni correction was applied to account for multiple comparisons.

### fMRI data analysis

All fMRI data were analyzed using the SPM12 software package.^3^ Data preprocessing included: (1) slice timing; (2) realignment of all EPI images to the first image; (3) coregistration of the anatomical image of the mean EPI image; (4) normalization of all images to MNI space; and (5) spatial smoothing (6-mm FWHM). The hemodynamic activity for all conditions (Auditory and Visual Rhythms, Auditory and Visual Controls, Rhythm Imagery) and six estimated movement parameters as regressors were first modeled within a general linear model (Friston et al., 1997) for each participant. In the second-level analysis, we carried out a random-effect ANOVA. Firstly, we focused on direct comparison between modalities and within each group. In both groups, we compared (1) Visual Rhythm to baseline, (2) Auditory Rhythm to baseline, and (3) Auditory Rhythm to Visual Rhythm. Next, we compared activation induced by Auditory Rhythm to Visual Rhythm across the groups (modality x group interaction). Then we focused on differences in brain activation induced by Visual and Auditory Rhythm compared to Visual Control and Auditory control. In both groups, we compared (1) Visual Rhythm to Visual Control and (2) Auditory Rhythm to Auditory Control. Subsequently, between-group analyses were performed to compare the activation induced by the experimental conditions (Visual Rhythm and Auditory Rhythm) to the control conditions (Visual Control and Auditory Control) in both groups. Moreover, we performed multiple regression analysis. Our participants results obtained in Visual Rhythm Tasks during the fMRI sessions were correlated with fMRI activation. In all contrasts, we applied a voxel-wise threshold of *p* < 0.001 uncorrected and *p* < 0.05 FWE threshold for the cluster extent. A probabilistic atlas of the human brain, as implemented in the SPM Anatomy Toolbox 2.2b (Eickhoff et al., 2005), was used to support the localization of the observed effects.

## Results

### Behavioral results

In the Auditory Rhythm task, the average performance level was *M* = 85.21%; SD = 6.82; (average from 3 days) in musicians. Non-musicians reached a performance point of an average of 73.66% (SD = 11.51; average 3 days). In the Visual Rhythm tasks, musicians performed above 80% (*M* = 80.26%; SD = 11.11; average 3 days), and non-musicians performed about 64% (*M* = 63.68%; SD = 13.28; average 3 days). Due to our results in the Adaptive Staircase Procedure, all participants’ responses were converted into the weighted arithmetic mean (raw mean result × number of items in the staircase/6 (the lower limit)). This method allows one to take into account the staircase result obtained by the participants in their final level of accuracy.

**Figure 2:**
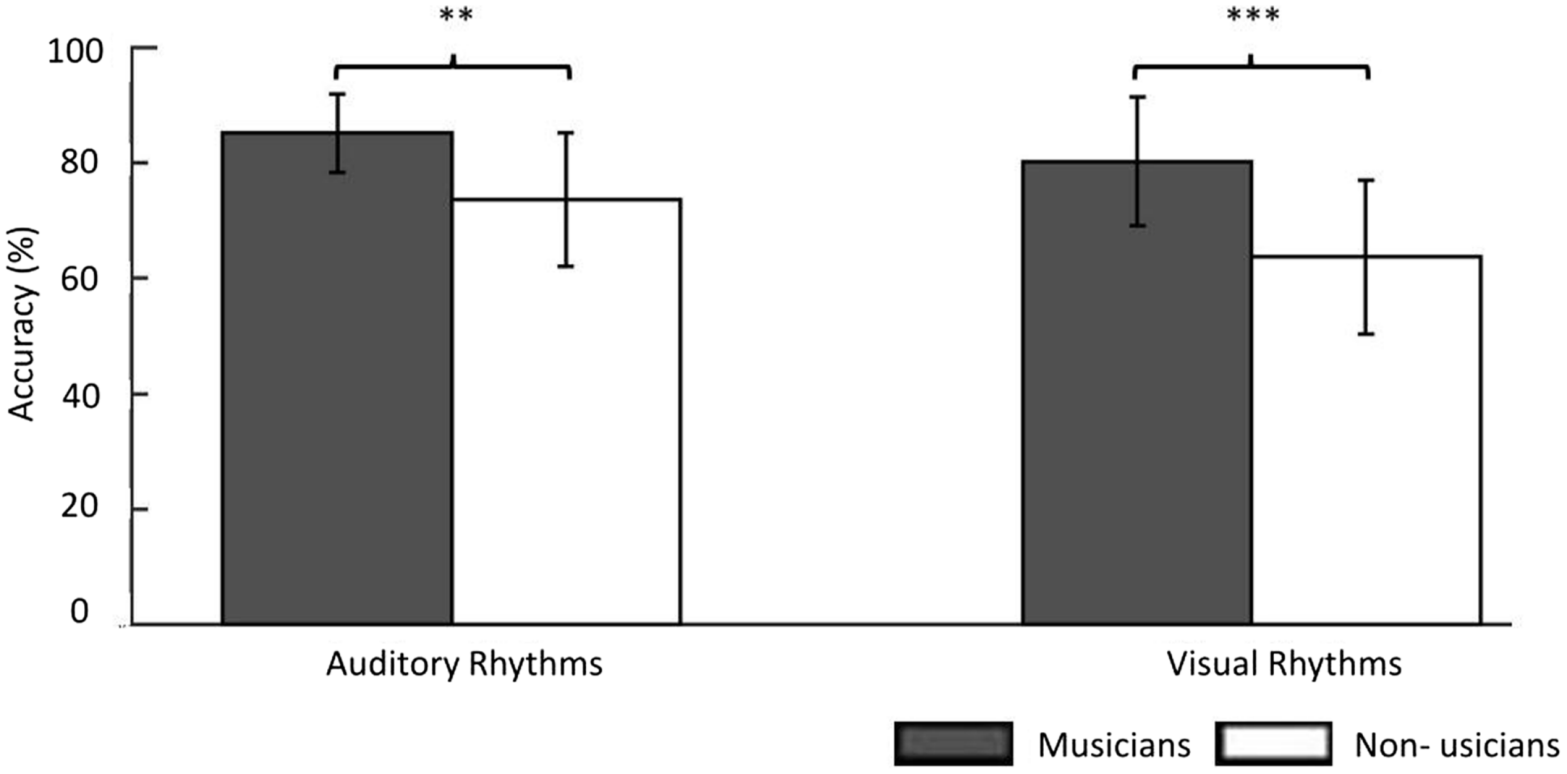
The behavioral results showed the accuracy level of the same/different decision in the experimental task. In the Auditory Rhythm task, the average performance level was 85% in musicians and 74% in non-musicians. In the Visual Rhythm tasks, musicians performed above 80% and non-musicians performed ~65%. For a better understanding this figure, we used results before changing into the weighted arithmetic mean. However, the results of the between-group comparisons in both tasks were based on a repeated-measures ANOVA (group × modality × day) in which we used the weighted arithmetic mean. Thresholds: ^**^p < 0.01, ^***^p < 0.001. Error bars represent S.E.M.

The three-way repeated-measure ANOVA (group × modality × day). The main interaction (group × modality × day) was insignificant (*F*(2, 35) = 1.72, *p* = 0.19) as well as the main effect of training (*F*(2, 72) =2.08, *p* = 0.13), interaction modality × day (*F*(2, 72) = 0.32, *p* = 0.73), and interaction group × day (*F*(2, 35) = 1.45, *p* = 0.25). However, the between-group effect was significant (*F*(1, 36) = 15.30, *p* < 0.001) as well as the interaction between group and modality proved significant (*F*(1, 36) = 4.81, *p* < 0.05). This means that we could not observe any effect of day-to-day training on accuracy in any of the groups, yet the between-group effect was present. Musicians showed significantly better performance than non-musicians in both tasks (Auditory Rhythm task *p* < 0.01; Visual Rhythm task *p* < 0.001; Figure 2).

Next, we tested whether there was any significant variance in performance during the fMRI session, for example, whether there were learning effects with better performance in the latter part of the session. To check performance changes during the scanning day in both groups, we performed three separate three-way repeated-measure ANOVAs with different sizes of temporal moving windows [group(2) × modality(2) × a moving trial window within the session (either 9 windows of 5 trials, 4 windows of 10 trials, or 3 windows of 15 trials)] on all participants’ results obtained during the fMRI session. There were 45 trials for both auditory and visual tasks during fMRI session. There was a main effect of 5-trial window *F*(8, 288) = 2.40, *p* = 0.02. However, this effect did not differ between groups: interaction of 5-trial window and group was insignificant *F*(8, 288) = 1.17, *p* = 0.32, and the three way-interaction (trial window × group × modality) was also insignificant *F*(8, 288) = 0.91, *p* = 0.51. The same result was obtained with 10-and 15-trial windows.

### fMRI results

**Figure 3:**
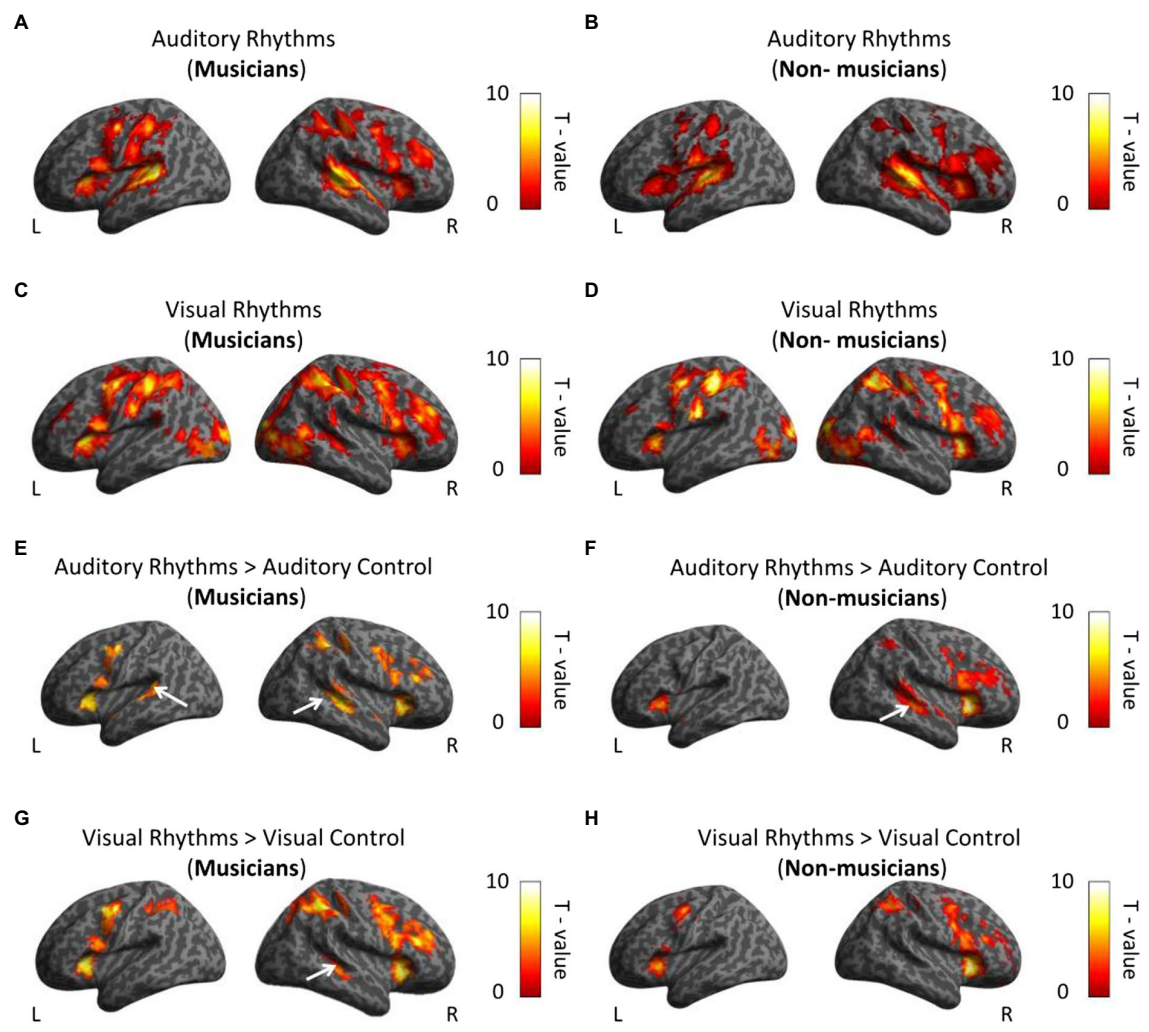
Whole-brain analysis. **(A,B)** Activations induced by Auditory Rhythm in musicians **(A)** and non-musicians **(B)**. In both groups, we found bilateral activations in frontal, parietal and temporal cortex **(C,D)** Activations induced by Visual Rhythms in musicians **(C)** and non-musicians **(D)**. In both groups, we found bilateral activations in frontal, parietal and occipital cortex. Moreover, we observed activation in right auditory cortex in the both group. **(E,F)** Activations induced by Auditory Rhythm relative to Auditory control in musicians **(E)** and non-musicians **(F)**. In both groups, we found activations in the auditory cortex. **(G,H)** Activations induced by Visual Rhythms relative to Visual Control in musicians **(G)** and non-musicians **(H)**. In musicians, we found statistically significant activation in the right dorsal auditory cortex (Middle Temporal Gyrus, *t* = 5.10). This comparison did not show any activation in the auditory cortex in non-musicians at this level of statistical significance. Thresholds: **(A–H)** *p* < 0.001 unc. voxel-wise, *p* < 0.05 FWE cluster-wise.

First, we compared the activation induced by the Auditory and the Visual Rhythm conditions with activation during rest periods in musicians and non-musicians. For the Auditory Rhythm condition, we observed bilateral activations in the frontal, parietal, and temporal cortex and the cerebellum in both subject groups (Figures 3A,B; Supplementary Table S1). The Visual Rhythm condition induced bilateral activations in the frontal, parietal and occipital cortex in both hemispheres, with similar patterns observed in both groups. Critically, in both musicians and non-musicians, the Visual Rhythm condition induced activation also in the right auditory cortex (Middle Temporal Gyrus; peak MNI: 48, −34, −1 *t* = 5.47 for musicians; peak MNI: 48, −28, −1, *t* = 3.62 for non-musicians; Figures 3C,D; Supplementary Table S2).

**Figure 4:**
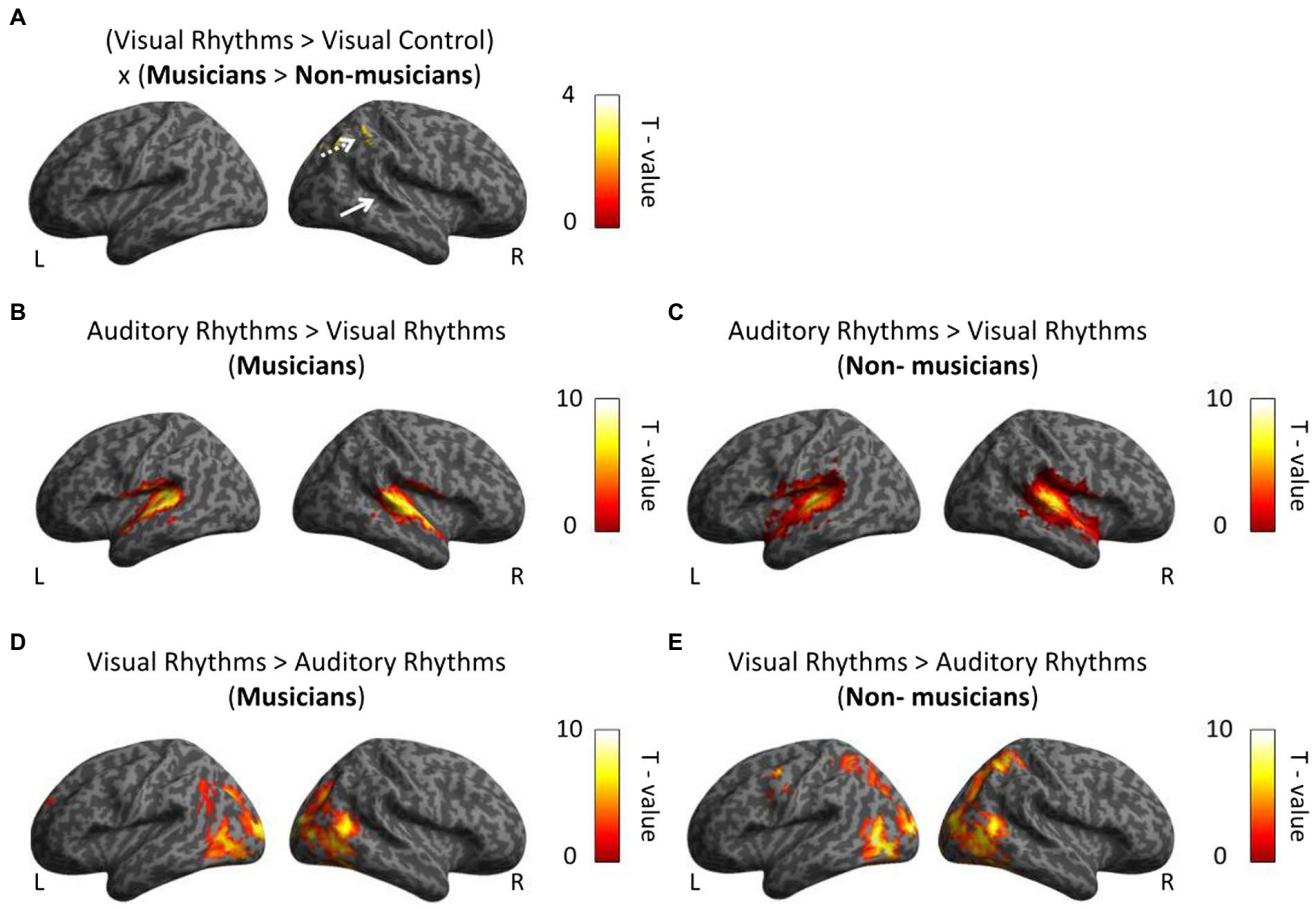
Whole-brain analysis. **(A)** In musicians, the right parietal lobe is associated with perceptual rhythm discrimination. White arrows indicate activation in the auditory cortex. **(B,C)** In both group we observed greater bilateral activation in auditory cortex in comparison between Auditory Rhythm vs. Visual Rhythm. **(D,E)** Visual Rhythm compared to Auditory Rhythm induced activation in occipital cortex in the both groups and parietal cortex in non-musicians. Thresholds: **(A–E)** *p* < 0.001 unc. Voxel-wise, *p* < 0.05 FWE cluster-wise.

To investigate whether the observed activations are specific to the rhythm discrimination tasks, we subsequently compared the activations induced by Auditory and Visual Rhythm conditions relative to Visual and Auditory control conditions in both subject groups. In musicians, the Auditory Rhythm vs. Auditory Control contrast revealed bilateral activation in the auditory cortex (right hemisphere: peak MNI: 48, −31, 2, *t* = 6.74; left hemisphere: peak MNI: −54, −40, 11, *t* = 5.70; Figure 3E; Supplementary Table S3). We found the same activation pattern in non-musicians (right hemisphere: peak MNI: 51, −25, −1, *t* = 6.70; left hemisphere: peak MNI: −51, 2, −1, *t* = 6.76; Figure 3F; Supplementary Table S3). We observed activations in the frontal, parietal, and insula cortex in both groups (Figures 3E,F; Supplementary Table S3). In the second comparison, we analyzed activation induced by Visual Rhythm relative to Visual Control. In musicians, we found activation in the right dorsal auditory cortex (Middle Temporal Gyrus, peak MNI: 48, −31, −4, *t* = 5.10; Figure 3G; Supplementary Table S4). This comparison did not show any activation in the auditory cortex in non-musicians at this level of statistical significance (Figure 3H; Supplementary Table S4). Additional activations were also found in the frontal and parietal regions in both groups (Figures 4G,H; Supplementary Table S4).

We then directly tested our hypothesis, that is, we asked whether musical training leads to stronger cross-modal activation of the auditory cortex in musicians for visual rhythm processing. To this aim, we performed a whole-brain interaction analysis including the data from the visual conditions and both groups (Visual Rhythm > Visual Control × musicians > non-musicians). Contrary to our task-specific sensory independent hypothesis, we did not find differences in activation within the auditory cortex (even at an exploratory voxel threshold of *p* < 0.01). Compared to non-musicians, musicians had significantly greater activation in the right parietal lobe (Angular gyrus, peak MNI: 39, −64, 47, *t* = 4.91; Figure 4A; Supplementary Table S5). We subsequently performed a similar whole-brain interaction analysis for the auditory conditions (Auditory Rhythm > Auditory Control × musicians > non-musicians). We and did not find any differences between groups in this analysis.

Finally, we directly compared activations induced by Auditory Rhythm to activation induced by Visual Rhythm. This control analysis was meant to test the level of overlap between pattern of activity for visual vs. auditory rhythm. Compared to Visual Rhythm, Auditory Rhythm resulted in greater activation of bilaterally auditory cortex (right hemisphere: peak MNI: 57, −19, 5, *t* = 13.05; left hemisphere: peak MNI: −57, −19, 8, *t* = 14.47) in musicians (Figure 4B; Supplementary Table S6). In non-musicians, we found a similar activation pattern, but we also observed greater activation in the cerebellum and occipital cortex during Auditory Rhythm task (Figure 4C; Supplementary Table S6). In the Visual Rhythm condition, compared to Auditory Rhythm condition, we observed greater activation in occipital cortex and frontal cortex in both groups (Figures 4D,E; Supplementary Table S7). In non-musicians, we also observed activation in the parietal cortex (Figure 4E; Supplementary Table S7).

Next, we tested for an interaction between the rhythm modality and the group (Auditory Rhythm > Visual Rhythm × musicians > non-musicians) to check whether musicians and non-musicians had different activation patterns for both types of rhythms. This comparison has not shown any differences in brain activity in any of the groups. Finally, to verify whether the between-group difference observed in the parietal cortex could be related to between-group differences in task performance, we performed a multiple regression analysis in which individual subjects’ behavioral results (mean scores) were correlated with fMRI activation. Two regressors: accuracy and subject group were included in the analysis. This analysis did not produce any significant results (the main effects and the interaction between groups and performance were insignificant), even at exploratory thresholds of *p* < 0.01 voxel-wise.

## Discussion

Our main aim was to investigate the brain mechanisms underlying how skills trained in the auditory modality (*via* musical training) may improve specific visual abilities (visual rhythm processing). Our hypothesis posited that the superior rhythm discrimination of musicians is related to the task-specific sensory independent reorganization of their auditory cortex (Amedi et al., 2017; Heimler and Amedi, 2020). In line with this interpretation, one should expect that the performance in the visual rhythm discrimination task would be related to activation in the auditory cortex. Our results, however, proved the contrary. We did not find greater task-related activation in the musicians’ group in the auditory cortex.

In our study, musicians significantly exceeded non-musicians in both the visual and auditory rhythm discrimination tasks. This finding is consistent with previous results. Professional musicians perform better than non-musicians in any task related to auditory rhythm, e.g., a memory (Schaal et al., 2015), a rhythm change detection task (Geiser et al., 2009), a rhythm reproduction task (Drake, 1993), or a finger tapping task (Franěk et al., 1991). Similarly to our results, Rammsayer et al. (2012) found that temporal information processing in the auditory and visual modality is more accurate in musicians than in non-musicians.

In our fMRI analysis, we first focused on comparisons between modalities in both groups to answer the question whether the rhythms presented in visual and auditory modalities were processed differently by the brain. In both groups, we observed massive bilateral activation in the frontal, parietal, and temporal cortex as well as in the cerebellum induced by the Auditory Rhythm condition. In the Visual Rhythm condition, we observed bilateral activation in the frontal, parietal and occipital cortex and in the right auditory cortex in both groups. Our findings are consistent with those of Grahn et al. (2011) and Karabanov et al. (2009), who showed similarity in neural correlates of visual and auditory rhythm production. In the Auditory Rhythm condition, compared to Visual Rhythm, we observed an increased activation only in the auditory cortex in both groups. This result is in contrast to previous study comparing musicians and non-musicians, in which musicians had greater activation in premotor cortex, cerebellum and supplementary motor area during auditory rhythm tasks (Grahn and Brett, 2007). This difference could be observed due to the fact that our auditory task was rather easy and the rhythm used in the task was not complicated for professional musicians as reflected by their behavioral results reaching ceiling in most of the cases. The overlap between modalities in the frontal and parietal cortex could suggest that auditory and visual rhythm perception activates a similar network of brain areas, which is in line with the findings of Schubotz et al. (2000). These results may suggest that musicians’ and non-musicians’ brains take advantage of the same general, modality-independent timing mechanism such as the hypothetical internal clock postulated by numerous studies (Rammsayer et al., 2012; Schaal et al., 2015).

In contrast to our results, several studies show different brain activation in musicians and non-musicians during visual or auditory rhythm perception (e.g., Chen et al., 2008; Grahn and Rowe, 2009; Lee and Noppeney, 2011). In some studies, synchronization with audio-visual rhythm enhanced neuronal activation in professional musicians’ brains in the postcentral gyrus, left superior temporal gyrus, insula, and cerebellum (Penhune et al., 1998; Hutchinson et al., 2003; Jongsma et al., 2004; Grahn and Rowe, 2009). The differences between this study and our study could stem from the fact that our tasks were rather perceptual and not as complex as those usually used in studies involving professional musicians. Furthermore, the relatively modest differences between musicians and non-musicians obtained in our study could stem from the fact that out of the three standard components of “musicality,” rhythmicity stands out as the most genetically heritable (de Manzano and Ullén, 2018; Kotz et al., 2018; Fiveash et al., 2021). Extensive musical instrument training might not influence neural mechanisms of rhythmicity as much as much as it influences other capacities.

Our hypothesis was based on the task-specific sensory independent organization of the cortex (Amedi et al., 2017; Heimler and Amedi, 2020) and assumed that after long-term musical training the auditory cortex can be reorganized. However, our results showed that the musicians do not exhibit the increased activation for the Visual Rhythm task in the auditory cortex. Instead, they display greater activation in the Inferior Parietal Lobe. This observation, therefore, indicates that the tasks-specific sensory independent hypothesis seems not to apply in the case of musical expertise and visual rhythm processing.

Learning to play an instrument is a highly complex and multimodal task which involves interaction of several brain areas and high-order cognitive functions (Herholz and Zatorre, 2012). Greater activation in musicians’ Inferior Parietal Lobe during Visual Rhythm task could perhaps be explained by the observation that this region had been activated by tasks based on temporal information (Penhune et al., 1998; Karabanov et al., 2009; Hove et al., 2013). Bilateral activations in the Inferior Parietal Lobule were induced by auditory and visual rhythm working memory tasks (Konoike et al., 2012). Damage to the IPL can impair rhythm processing (Peretz, 1990; Di Pietro et al., 2004), and this region is crucial for retaining temporal information (Ravizza et al., 2004). The main limitation of our study was the lack of more challenging rhythm tasks such as production of rhythm, or tapping in order to check musicians’ rhythm abilities more widely and to compare activations in the brain induced by different tasks presented in visual and auditory modalities. Incorporating such tasks in the future studies could shed more light on the hypothesis about the existence of general, multimodal internal clock and brain reorganization after long-term musical training.

In conclusion, our results show that the musicians’ superior rhythm discrimination is most likely not related to cross-modal recruitment of the auditory cortex.

## Data availability statement

The raw data supporting the conclusions of this article will be made available by the authors, without undue reservation.

## Ethics statement

The studies involving human participants were reviewed and approved by The Committee for Research Ethics of the Institute of Psychology of Jagiellonian University. The patients/participants provided their written informed consent to participate in this study.

## Author contributions

MK, MZ, ŁB, and MS: designed research. MK, MZ, and MS: performed research, analyzed data, and wrote the manuscript. All authors contributed to the article and approved the submitted version.

## Funding

This work was supported by the Polish National Science Centre (grant number 2018/30/A/HS6/00595) to MS.

## Conflict of interest

The authors declare that the research was conducted in the absence of any commercial or financial relationships that could be construed as a potential conflict of interest.

## Publisher’s note

All claims expressed in this article are solely those of the authors and do not necessarily represent those of their affiliated organizations, or those of the publisher, the editors and the reviewers. Any product that may be evaluated in this article, or claim that may be made by its manufacturer, is not guaranteed or endorsed by the publisher.

## Footnotes

^1^ https://www.neurobs.com/

^2^ http://www.psimagopro.pl/index_pl.html

^3^ https://www.fil.ion.ucl.ac.uk/spm/software/spm12/
