# Supplementary material for: Superior visual rhythm discrimination in expert musicians is most likely not related to cross-modal recruitment of the auditory cortex
Source: Front Psychol. 2022 Oct 20;13:1036669. doi: 10.3389/fpsyg.2022.1036669 (PMC9632485; doi:10.3389/fpsyg.2022.1036669)
Supplement: Supplementary file 1 [file Data_Sheet_1.PDF]

Table 1. Activation for Auditory Rhythm task in the musicians and the non-musicians.

Anatomical region	BA	Cluster size (voxels)	MNI-coordinates X	Y	Z	t-value
Auditory Rhythms (Musicians)						
L superior temporal gyrus	1	3929	-57	-19	8	16.75
L superior temporal gyrus	22		-54	-37	11	15.41
L superior temporal gyrus	41		-66	-25	8	14.42
L posterior-medial frontal	NA	764	0	2	62	16.43
L posterior-medial frontal	8		3	17	50	10.48
R midcingulate cortex	8		9	23	32	4.92
R superior temporal gyrus	22	3998	60	-34	11	13.53
R superior temporal gyrus	41		57	-16	5	13.08
R superior temporal gyrus	22		54	-31	2	11.97
R cerebelum	NA	238	27	-58	-22	11.47
L cerebelum	NA		-21	-49	-25	5.33
cerebellar vermis	NA		6	-70	-13	5.32
Auditory Rhythms (Non-musicians)						
L superior temporal gyrus	40	10555	-51	-22	8	22.79
L superior temporal gyrus	41		-39	-31	11	22.46
R superior temporal gyrus	41		51	-31	11	20.35
R Heschls grys	41		45	-22	8	20.05
R superior temporal gyrus	22		63	-19	2	19.01
L superior temporal gyrus	41		-45	-19	2	18.14
L superior temporal gyrus	41		-63	-19	8	16.88
L cerebellum	NA		-24	-61	-22	16.80
R superior temporal gyrus	41		66	-22	11	15.97
L superior temporal gyrus	22		-51	2	-4	15.85
L superior temporal gyrus	22		-60	-31	14	15.53
R cerebellum	NA		24	-58	-22	14.81
R superior temporal gyrus	22		54	-1	-4	14.76
R temporal pole	NA		51	2	-7	14.62
R superior temporal gyrus	22		51	-10	-4	14.35
L postcentral gyrus	1		-60	-16	20	14.33
R postcentral gyrus	1	417	48	-28	50	8.64
R inferior parietal lobule	40		45	-40	53	6.39
R inferior parietal lobule	7		39	-43	47	6.23

NA. not appicable. Thresholds: $p < 0.001$ unc. voxel-wise. $p < 0.05$ FWE cluster-wise.

Table 2. Activation for Visual Rhythm task in the musicians and the non-musicians.

Anatomical region	BA	Cluster size (voxels)	MNI-coordinates X	Y	Z	t-value
Visual Rhythms (Musicians)						
L posterior-medial frontal	NA	13388	0	2	62	15.65
R supramarginal gyrus	40		42	-37	44	13.42
R cerebellum	NA		33	-55	-25	12.45
L precentral gyrus	6		-45	-4	50	12.19
L inferior parietal lobule	40		-45	-37	44	12.04
R insula lobe	45		-30	20	8	11.96
L posterior-medial frontal	NA		-9	8	50	11.90
R cuneus	18		18	-100	11	11.80
L putamen	NA		-21	-1	8	11.77
R postcentral gyrus	1		48	-28	44	11.61
R cerebellum	NA		27	-58	-22	11.61
R calcarine gyrus	17		15	-91	-4	11.56
R middle occipital gyrus	NA		36	-79	5	11.49
	NA		-21	-1	17	11.47
R superior occipital gyrus	18		24	-94	14	11.40
R fusiform gyrus	18		27	-82	-7	11.30
L middle frontal gyrus	10	188	-39	47	23	6.39
L inferior frontal gyrus	NA		-33	29	26	6.30
L middle frontal gyrus	9		-42	38	29	5.75
Visual Rhythms (Non-musicians)						
L postcentral gyrus	1	10046	-45	-31	50	13.75
R cerebelum	NA		30	-58	-22	13.66
R superior parietal lobule	7		33	-52	56	13.63
L middle occipital gyrus	18		-21	-94	11	13.41
L postcentral gyrus	1		-60	-16	23	13.20
L cerebellum	NA		-30	-55	-25	13.16
L cerebellum	NA		-27	-64	-22	13.15
	13		33	20	2	13.03
R posterior-medial frontal	6		6	11	53	12.72
R precentral gyrus	6		51	8	35	12.55
L posterior-medial frontal	6		-3	-1	62	12.41
R inferior parietal lobule	NA		30	-49	50	12.28
R postcentral gyrus	1		48	-28	50	12.01
L precentral gyrus	6		-45	-4	50	11.92
	13		-30	14	5	11.90
L putamen	25		-24	-1	11	11.86

NA. not appicable. Thresholds: $p < 0.001$ unc. voxel-wise. $p < 0.05$ FWE cluster-wise.

Table 3. Activation for Auditory Rhythm task relative to Auditory Control task in the musicians and the non-musicians.

Anatomical region	BA	Cluster size (voxels)	MNI-coordinates X	Y	Z	t-value
Auditory Rhythms vs. Auditory Control (Musicians)						
L posterior- medial frontal gyrus	NA	336	0	2	62	9.10
R posterior- medial frontal gyrus	8		6	17	50	7.11
R anterior cingulate cortex	8		9	26	29	3.73
L precentral gyrus	6	592	-51	-4	50	7.59
L insula	13		-33	20	5	7.17
L superior temporal gyrus	22		-57	5	-4	5.33
R insula	13	279	39	20	2	7.12
R temporal pole	38		57	11	-10	4.79
R superior temporal gyrus	22		51	-4	-4	3.42
R supramarginal gyrus	40	361	42	-37	44	6.94
R inferior parietal lobule	7		39	-46	53	5.03
R postcentral gyrus	1		45	-37	62	4.32
R superior temporal gyrus	22	242	48	-31	2	6.74
R inferior frontal gyrus	44	290	45	8	26	5.86
R precentral gyrus	6		51	2	47	5.36
R precentral gyrus	8		39	5	32	4.92
R middle frontal gyrus	9	156	45	32	29	5.75
L middle temporal gyrus	22	158	-54	-40	11	5.70
L superior temporal gyrus	2		-63	-28	5	4.96
L superior temporal gyrus	NA		-66	-34	11	4.63
Auditory Rhythms vs. Auditory Control (Non-musicians)						
R insula	13	1654	33	20	2	11.74
R inferior frontal gyrus	44		45	11	20	6.81
R superior temporal gyrus	22		51	-25	-1	6.70
R posterior-medial frontal gyrus	6	465	6	14	53	8.81
L posterior-medial frontal gyrus	6		-3	2	62	7.49
L posterior-medial frontal gyrus	NA		-9	11	50	6.53
L insula	13	251	-30	23	2	7.87
L insula	44		-33	14	14	5.78
L superior temporal gyrus	22	83	-51	2	-7	6.76
R inferior parietal lobule	40	122	48	-34	53	4.49
R inferior parietal lobule	7		42	-43	50	4.35
R superior parietal lobule	7		30	-49	44	4.16

NA. not appicable. Thresholds: $p < 0.001$ unc. voxel-wise. $p < 0.05$ FWE cluster-wise.

Table 4. Activation for Visual Rhythms tasks relative to Visual Control tasks in the musicians and the non-musicians.

Anatomical region	BA	Cluster size (voxels)	MNI-coordinates X	Y	Z	t-value
Visual Rhythms vs Visual Control (Musicians)						
L posterior-medial frontal	6	5357	-3	2	62	9.50
L insula	13		-30	23	8	8.52
L precentral gyrus	6		-51	-4	50	8.28
R supramarginal gyrus	40	895	42	-37	44	8.23
R inferior parietal lobule	7		33	-49	44	7.10
R inferior parietal lobule	7		39	-46	53	7.05
L inferior parietal lobule	40	303	-48	-37	44	5.80
L supperior parietal lobule	NA		-27	-46	41	4.75
L inferior parietal lobule	40		-36	-49	50	4.60
R middle temporal gyrus	21	86	48	-31	-4	5.10
R superior temporal gyrus	22		48	-40	8	3.46
Visual Rhythms vs. Visual Control (Non-musicians)						
R insula	13	2562	33	20	2	11.59
L insula	13		-30	20	2	8.02
R precentral gyrus	6		51	8	35	7.90
L posterior-medial frontal gyrus	8	745	3	20	47	9.61
L posterior-medial frontal gyrus	NA		-9	11	50	6.43
L suplementary motor cortex	NA		-12	20	32	6.03
L cerebelum (VI)	NA	270	-27	-58	-25	7.72
R cerebelum (VI)	NA		24	-58	-22	6.43
L cerebelum (VI)	NA		-6	-73	-16	5.65
L precentral gyrus	6	262	-48	-4	47	7.57
L precentral gyrus	44		-60	8	20	4.78
L precentral gyrus	Na		-33	-7	50	4.42
R inferior frontal gyrus	9	249	42	32	29	6.93
R middle frontal gyrus	NA		33	41	14	4.92
R supperior orbital gyrus	47		27	44	-13	4.80
R supperior parietal lobule	7	436	30	-49	44	6.62
R inferior parietal lobule	40		42	-40	53	6.13
R supramarginal gyrus	40		45	-34	44	5.18
R ventral	NA	98	3	-22	-10	5.19
R ventral	NA		9	-19	-16	5.07
L ventral	NA		-9	-22	-16	4.79

NA. not appicable. Thresholds: $p < 0.001$ unc. voxel-wise. $p < 0.05$ FWE cluster-wise.

Table 5. Interaction between the tasks and the groups (Visual Rhythms vs. Visual Control) x (Muscians vs. Non-musicians)

Anatomical region	BA	Cluster size (voxels)	MNI-coordinates X	Y	Z	t-value
Interaction: (Visual Rhythms vs. Visual Control) x (Musicians vs. Non-musicians)						
R angular gyrus	39	135	39	-64	47	4.91
R inferior parietal lobule	7		42	-49	53	4.19
R supramarginal gyrus	40		45	-40	44	3.77

NA. not appicable. Thresholds: $p < 0.001$ unc. voxel-wise. $p < 0.05$ FWE cluster-wise.

Table 6. Activation for Auditory Rhythms vs. Visual Rhythms in the musicians and the non-musicians.

Anatomical region	BA	Cluster size (voxels)	MNI-coordinates X	Y	Z	t-value
Auditory Rhythms > Visual Rhythms (Musicians)						
L superior temporal gyrus	1	1081.00	-57	-19	8	14.47
L superior temporal gyrus	40		-45	-28	11	13.02
L superior temporal gyrus	41		-66	-25	8	12.87
R superior temporal gyrus	41	1019.00	57	-19	5	13.05
R superior temporal gyrus	41		54	-7	-1	12.80
R superior temporal gyrus	41		66	-22	8	12.57
Auditory Rhythms > Visual Rhythms (Non-musicians)						
L superior temporal gyrus	41	1675.00	-39	-31	11	22.18
L superior temporal gyrus	40		-51	-22	8	21.75
L superior temporal gyrus	41		-45	-19	2	17.97
R superior temporal gyrus	41	1782.00	51	-31	11	20.08
R Heschl gyrus	41		45	-22	8	19.90
R superior temporal gyrus	22		63	-19	2	17.76
	NA	851.00	18	-25	-7	5.78
	NA		-3	-37	-10	5.20
cerebellar vermis	NA		3	-64	-4	5.00

NA. not appicable. Thresholds: $p < 0.001$ unc. voxel-wise. $p < 0.05$ FWE cluster-wise.

Table 7. Activation for Visual Rhythms vs. Auditory Rhythms in the musicians and the non-musicians.

Anatomical region	BA	Cluster size (voxels)	MNI-coordinates X	Y	Z	t-value
Visual Rhythms vs Auditory Rhythms (Musicians)						
R cuneus	18	2517	18	-97	11	8.54
R calcarine gyrus	17		15	-91	-4	8.21
R superior occipital gyrus	18		24	-94	17	8.13
R precunes	23	256	9	-52	26	4.52
L precuneus	31		-6	-58	23	4.39
R precunes	23		3	-49	20	4.35
L superior medial gyrus	9	266	-3	50	29	4.06
R superior medial gyrus	10		9	65	26	3.98
L superior medial gyrus	9		0	53	38	3.97
Visual Rhythms vs. Auditory Rhythms (Non-musicians)						
L middle occipital gyrus	18	891	-21	-94	11	8.73
L middle occipital gyrus	19		-42	-64	2	8.39
L inferior occipital gyrus	19		-39	-73	-4	7.52
R superior parietal lobule	7	1666	33	-52	56	7.26
R superior occipital gyrus	18		24	-88	11	7.13
R middle temporal gyrus	19		45	-64	5	7.04
L precentral gyrus	6	86	-39	-7	44	6.19
L precentral gyrus	6		-45	-4	35	4.43
L middle occipital gyrus	NA	287	-27	-70	26	4.85
L superior parietal lobule	7		-27	-55	56	4.82
L superior parietal lobule	7		-24	-64	47	4.69

NA. not appicable. Thresholds: $p < 0.001$ unc. voxel-wise. $p < 0.05$ FWE cluster-wise.
